# High Mobility Group Box 1 Promotes Lung Cancer Cell Migration and Motility via Regulation of Dynamin-Related Protein 1

**DOI:** 10.3390/ijms22073628

**Published:** 2021-03-31

**Authors:** Wei-Lun Liu, Chia-Yang Li, Wei-Chung Cheng, Chia-Yuan Chang, Yung-Hsiang Chen, Chi-Yu Lu, Shu-Chi Wang, Yu-Ru Liu, Meng-Hsuan Cheng, Inn-Wen Chong, Po-Len Liu

**Affiliations:** 1 School of Medicine, College of Medicine, Fu Jen Catholic University, New Taipei City 242, Taiwan; medrpeterliu@gmail.com; 2 Division of Critical Care Medicine, Department of Emergency and Critical Care Medicine, Fu Jen Catholic University Hospital, Fu Jen Catholic University, New Taipei City 243, Taiwan; 3 Graduate Institute of Medicine, College of Medicine, Kaohsiung Medical University, Kaohsiung 807, Taiwan; chiayangli@kmu.edu.tw; 4 Center for Cancer Research, Kaohsiung Medical University, Kaohsiung 807, Taiwan; shuchiwang@kmu.edu.tw; 5 Graduate Institute of Biomedical Science, Research Center for Cancer Biology, China Medical University, Taichung 404, Taiwan; wccheng@mail.cmu.edu.tw; 6 Department of Mechanical Engineering, National Cheng Kung University, Tainan 701, Taiwan; cychang0829@gmail.com; 7 Graduate Institute of Integrated Medicine, College of Chinese Medicine, China Medical University, Taichung 404, Taiwan; yhchen@mail.cmu.edu.tw; 8 Department of Psychology, College of Medical and Health Science, Asia University, Taichung 413, Taiwan; 9 Department of Biochemistry, College of Medicine, Kaohsiung Medical University, Kaohsiung 807, Taiwan; cylu@kmu.edu.tw; 10 Department of Medical Laboratory Science and Biotechnology, Kaohsiung Medical University, Kaohsiung 807, Taiwan; 11 Center for Liquid Biopsy, Kaohsiung Medical University, Kaohsiung 807, Taiwan; 12 Department of Respiratory Therapy, College of Medicine, Kaohsiung Medical University, Kaohsiung 807, Taiwan; lu6525@ms42.hinet.net; 13 Division of Pulmonary and Critical Care Medicine, Department of Internal Medicine, Kaohsiung Medical University Hospital, Kaohsiung 807, Taiwan; 14 School of Medicine, College of Medicine, Kaohsiung Medical University, Kaohsiung 807, Taiwan; 15 Department of Medical Research, Kaohsiung Medical University Hospital, Kaohsiung Medical University, Kaohsiung 807, Taiwan

**Keywords:** non-small cell lung cancer, high mobility group box 1, dynamin related protein 1, cytoskeleton dynamics, lamellipodia/filopodia, mitochondrial fission

## Abstract

High mobility group box 1 (HMGB1) has been demonstrated to promote the migration and invasion of non-small cell lung cancer (NSCLC). However, the mechanism of action of HMGB1 in regulating tumor mobility remains unclear. Therefore, we aimed to investigate whether HMGB1 affects mitochondria distribution and regulates dynamin-related protein 1 (DRP1)-mediated lamellipodia/filopodia formation to promote NSCLC migration. The regulation of mitochondrial membrane tension, dynamics, polarization, fission process, and cytoskeletal rearrangements in lung cancer cells by HMGB1 was analyzed using confocal microscopy. The HMGB1-mediated regulation of DRP1 phosphorylation and colocalization was determined using immunostaining and co-immunoprecipitation assays. The tumorigenic potential of HMGB1 was assessed in vivo and further confirmed using NSCLC patient samples. Our results showed that HMGB1 increased the polarity and mobility of cells (mainly by regulating the cytoskeletal system actin and microtubule dynamics and distribution), promoted the formation of lamellipodia/filopodia, and enhanced the expression and phosphorylation of DRP1 in both the nucleus and cytoplasm. In addition, HMGB1 and DRP1 expressions were positively correlated and exhibited poor prognosis and survival in patients with lung cancer. Collectively, HMGB1 plays a key role in the formation of lamellipodia and filopodia by regulating cytoskeleton dynamics and DRP1 expression to promote lung cancer migration.

## 1. Introduction

With advancements in medical diagnosis, the survival rate of cancer patients has improved, except for that of lung cancer, where late diagnosis is the primary reason for a five-year survival rate of less than 5% [1]. Though low-dose computed tomography (LDCT) screening has improved the five-year survival rate of patients with lung cancer, the usage of LDCT is not widely promoted [2]. Advanced lung cancers have high metastatic activity that results in distinct metastasis [3]. Therefore, in addition to the requirement of early diagnosis, the inhibition of the metastasis and invasion of lung cancer is a critical issue in therapy currently.

High mobility group box 1 (HMGB1) with a genetic location on the 13q12.3 chromosome belongs to the high mobility group box superfamily. It is a DNA-binding protein that regulates several cellular processes, such as inflammation, cell differentiation, and tumor cell migration [4]. In the nucleus, HMGB1 acts as a transcription factor and serves many DNA-dependent nuclear processes, including transcription, replication, recombination, and repair [5]. In the intracellular compartments, HMGB1 exists in endosomes [6], exosomes [7], endoplasmic reticulum–Golgi intermediate compartments [8], and cell surfaces [3]. In the extracellular compartments, HMGB1 mediates cell proliferation, differentiation, tissue regeneration, inflammation/immune responses, and tumor progression [9,10]. A previous study indicated that HMGB1 plays a critical role in tumor development, epithelial–mesenchymal transition, and the prognosis of lung cancer [11]. Furthermore, HMGB1 was shown to increase matrix metalloproteinase-9 expression through the phosphatidylinositol 3-kinase/protein kinase B and nuclear factor kappa-light-chain-enhancer of activated B cells signaling pathways, and it was found to promote lung cancer migration, invasion, and metastasis [12].

Mesenchymal cell motility is characterized by a polarized distribution of actin filaments. The leading-edge protrusions of migrating cells are formed by both sheet-like lamellipodia and rod-like filopodia [13], which are composed of meshworks and bundles of actin filaments [14]. The driving force of lamellipodia and filopodia is the barbed end (plus end) elongation formed by the polymerization of actin filaments toward the plasma membrane—a process that pushes the cell edge forward and is the key step in cell migration [15]. Increased filopodia formation has been indicated to promote migration [16]. The HMGB1/receptor for advanced glycation end products (RAGE) axis facilitates neural stem cell migration through filopodia formation [17]. In addition, abundant filopodia have been demonstrated as a characteristic feature of invasive carcinoma cells [18]. Though these studies have indicated that HMGB1 regulates filopodia formation and controls cancer cell migration, the mechanism of action has not been well-investigated.

Mitochondrial trafficking has emerged as a central regulator of metastatic competence in disparate tumors [19]. A recent study showed that cortical mitochondria supported membrane lamellipodia dynamics and actin cytoskeleton remodeling, resulting in increased cancer cell motility and invasion [20]. Cortical mitochondria produce ATP, which provides an efficient “regional” energy source to fuel membrane–cytoskeletal dynamics to regulate lamellipodia and filopodia formation [19]. In addition, a previous study showed that patients with highly phosphorylated dynamin-related protein 1 (DRP1) were associated with an increased risk of tumor relapse after neoadjuvant chemoradiotherapy [21]. Furthermore, HMGB1 induces DRP1-dependent mitochondrial dynamics via the RAGE–extracellular-signal-regulated kinase (ERK) signaling pathway, which is critical for autophagy and chemoresistance [21]. However, the correlation between HMGB1 and DRP1 in lung cancer and the way that HMGB1 drives mitochondrial fission to control cell migration and mobility have not been explored. Therefore, in this study, we aimed to evaluate the mechanism by which HMGB1 regulates motility and migration during lung cancer metastasis.

## 2. Results

### 2.1. HMGB1 Regulates Membrane Tension, Dynamics, Polarization, and Cytoskeletal Rearrangements in Lung Cancer Cells

**Figure 1:**
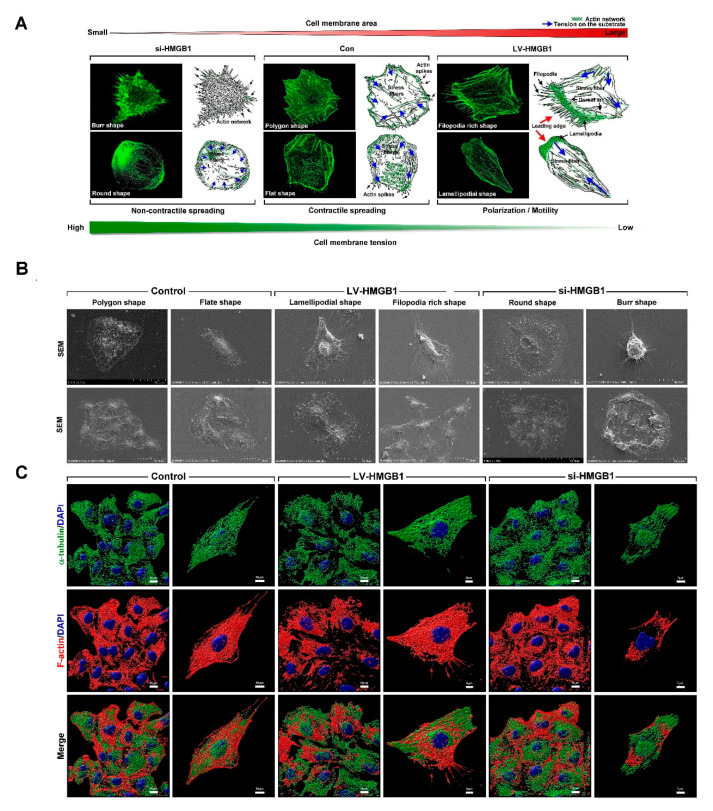
HMGB1 regulates lung cancer cell spreading and polarization through the regulation of microtubule and actin dynamics. (**A**) A549 cells were transfected with LV-HMGB1 or small interfering (si)-HMGB1 for 48 h, and then they were stained with F-actin and analyzed using confocal microscopy and the Imaris software (red arrows: leading edge; blue arrows: stress fibers. (**B**) Cell morphology and polarity were examined by SEM analysis. (**C**) To determine the organization of actin and microtubule cytoskeleton in cellular protrusions and polarities, the cells were stained with F-actin (red color), α-tubulin (green color), and nucleus dye (blue color), and they were analyzed using confocal microscopy and the Imaris software. Data are representative of three independent experiments. HMGB1: high mobility group box 1; si-HMGB1: HMGB1 small interfering RNA; Con: control; LV-HMGB1: lentiviral vector-carrying HMGB1; DAPI: 4′,6-diamidino-2-phenylindole.

HMGB1 is a regulator of cell initiation, polarity, motility, and metastasis [22,23]. To determine whether HMGB1 expression affects spreading and polarization in lung cancer, human lung cancer A549 cells were infected with lentiviral vector-carrying HMGB1 (LV-HMGB1) or transfected with small interfering (si)-HMGB1 for 48 h. The changes in cell morphology were examined by F-actin immunostaining and imaging using confocal microscopy, followed by 3D image construction using the Imaris software. Our results showed that in the control group, cells presented as polygon and flat shapes; they transitioned from the non-contractile spreading phase into the contractile spreading phase and had a larger cell surface area and lower cell membrane tension (Figure 1A). Migration was found to be a polarized cellular process that moved from a protrusive front edge to a retracting trailing edge. In the group with HMGB1 overexpression, cells presented with a filopodia-rich shape and a lamellipodial shape with a high membrane area, lower membrane tension, and higher polarity and motility (Figure 1A). Taken together, these findings indicate that HMGB1 might play a critical role in the regulation of membrane tension, dynamics, and polarization in lung cancer cells. The silencing of HMGB1 expression resulted in a small cell membrane area, a high membrane tension, and non-contractile spreading. There were two types of cell shapes: burr-shaped and round-shaped. The burr-shaped cells were small and had serrated edges over the entire surface with a high membrane tension and less surface area adhered to the cell matrix. The round-shaped cells revealed weak cell substrate attachment, although they had higher membrane tension than the interphase cells, and membrane folds were clearly visible at this stage. From the front to the rear, actin-mediated forces sequentially promoted cell protrusion, adhesion, contraction, and retraction. Microtubule network polarity controls the establishment and maintenance of the spatial and temporal coordination of migration events and is therefore the key to persistent directed migration [24]. We further examined the effects of HMGB1 in regulating lamellipodia and filopodia formation in lung cancer cells by SEM analysis. Our data showed that in the LV-HMGB1 group, cell appearance revealed a lamellipodium/filopodium shape (Figure 1B). In addition, we further explored whether HMGB1 affected cell protrusions and polarity through the regulation of cytoskeletal microtubules and F-actin using immunofluorescence staining. Our data showed that in the control group, microtubules were distributed in the cytoplasm and showed alignment and directionality, while actin formed bundles, stress-fibers, and actin cortexes to maintain cell morphology. In the LV-HMGB1 group, microtubules were distributed in lamellipodia and had alignment and directionality, while actin formed filopodia and cortical actin around the cell nucleus. In the si-HMGB1 group, microtubules formed a network of evacuated patterns distributed in the cytoplasm. In addition, actin formed a large amount of cortexes around the cell (Figure 1C). These findings indicate that HMGB1 promotes lamellipodia and filopodia formation through the regulation of microtubule and actin dynamics.

### 2.2. HMGB1 Regulates Mitochondrial Dynamics and Increases Mitochondrial Fission in Lung Cancer Cells

**Figure 2:**
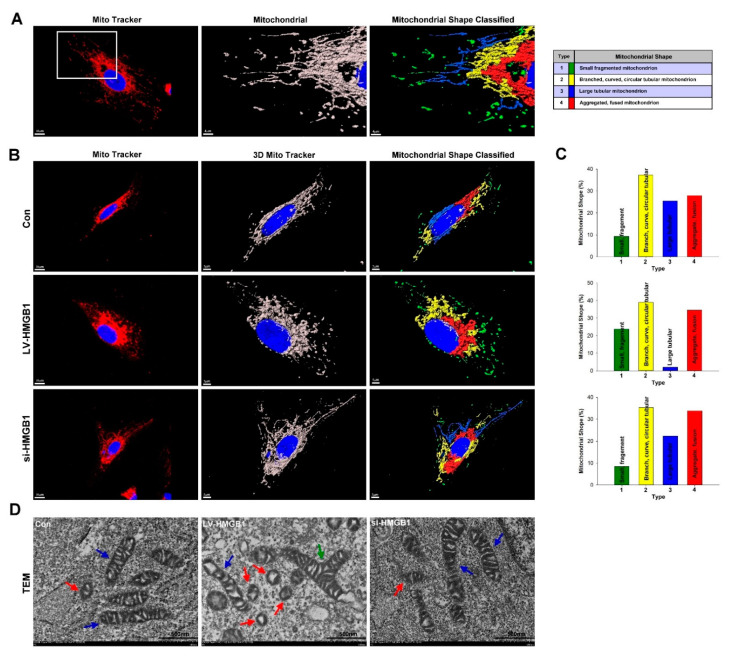
Mitochondrial dynamics is regulated by HMGB1. (**A**) Mitochondria were stained using the mitochondria tracker dye (red color) and nucleus dye (blue color) and analyzed using confocal microscopy and the Imaris software. Mitochondrial shapes (highlighted in white square as an example) were classified as type 1 (small fragmented mitochondria), type 2 (branched, curve, circular, and tubular mitochondria), type 3 (large tubular mitochondria), and type 4 (aggregated and fusion mitochondria). (**B**,**C**) A549 cells were transfected with LV-HMGB1 or si-HMGB1, respectively, for 48 h. The types of mitochondrial shapes were classified and quantified using confocal microscopy, the Imaris software, and the Mountains 8 software. (**D**) TEM analysis was used to confirm mitochondrial shapes (blue arrows: large mitochondria; red arrows: small fragmented mitochondria; green arrows: branched mitochondria).

Mitochondria exist as dynamic networks that often change size and subcellular distribution, and these dynamics are maintained by two opposing processes: fission and fusion [25]. Next, we examined the regulation of mitochondrial dynamics by HMGB1. The mitochondrial shapes were classified into type 1 (green color, fission mitochondria, and small fragmented), type 2 (yellow color, fission mitochondrial formation, branch, curve, and circular tubular mitochondria), type 3 (blue color, fusion mitochondria, and large tubular), and type 4 (red color, fusion mitochondria, and large fusion aggregate; see Figure 2A. Mitochondrial fission and small fragment formation were markedly increased in the LV-HMGB1 group compared to other groups. Large and long mitochondria (mitochondrial fusion) were notably increased in the control and si-HMGB1 groups (Figure 2B,C). Next, we examined the mitochondrial morphology by TEM analysis (Figure 2D). Our data revealed that LV-HMGB1 increased small and branch shaped mitochondria formation. This data indicated that HMGB1 regulated mitochondrial dynamics and increased the fission process.

### 2.3. HMGB1 Regulates DRP-1 Phosphorylation, Mitochondrial Dynamics, and Increases Mitochondrial Trafficking to the Leading Edge of Lung Cancer Cells

**Figure 3:**
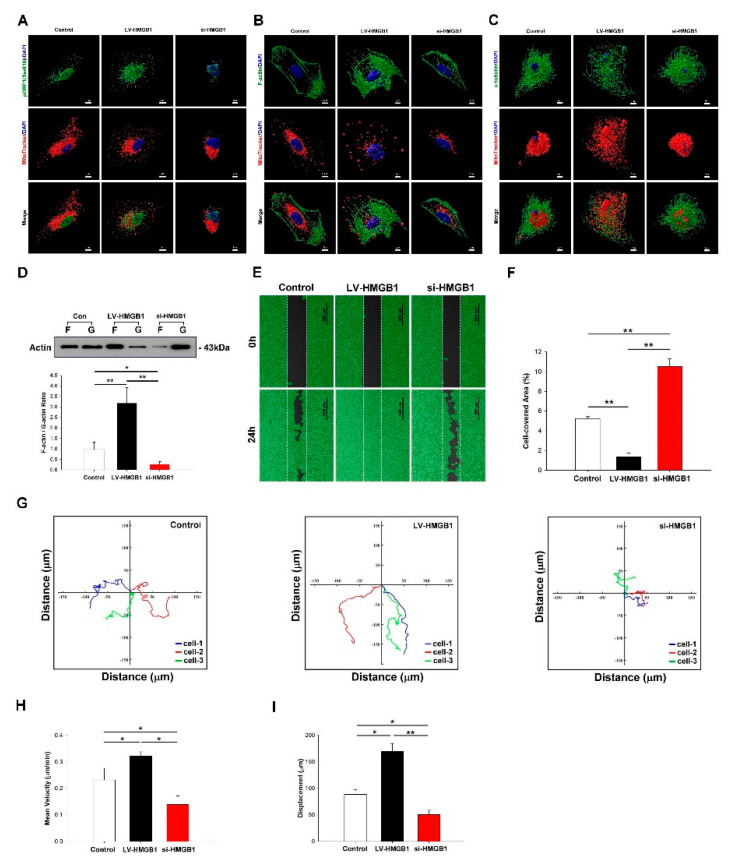
HMGB1 regulates DRP1 phosphorylation and mediates mitochondrial trafficking from the cortical cytoskeleton to the leading edge to promote cancer migration. (**A**) A549 cells were transfected with LV-HMGB1 or si-HMGB1 for 48 h. Cells were stained with HMGB1 (green color), phospho-DRP1 (pDRP1) (Ser616) (red color), and nucleus dye (blue color), and they were analyzed using confocal microscopy and the Imaris software. (**B**,**C**) Mitochondrial repositioning (mitochondria tracker dye: red color) and F-actin/α-tubulin (green color) expression, respectively, were analyzed using confocal microscopy and the Imaris software. (**D**) F-actin and G-actin expressions were analyzed by Western blot (WB). (**E**,**F**) Cell migration was analyzed using the wound-healing assay (scale bar: 500 μm). Cells were analyzed by time-lapse microscopy. (**G**) migration distance, (**H**) mean velocity, and (**I**) displacement were determined. * *p* < 0.05 and ** *p* < 0.01 compared with the control. F: F-actin; G: G-actin.

The upregulation of mitochondrial fission and the increased expression of DRP1 have been reported to promote cancer metastasis [26]. In migrating cancer cells, mitochondria localize at the leading edge along microtubules to meet high-energy demand and provide necessary supply characteristics [27]. Since HMGB1 increased mitochondrial fission, we further examined whether HMGB1 affected DRP1 expression and mitochondria shape. Our experimental results showed that LV-HMGB1 increased the expression of phospho-DRP1 (pDRP1) (Ser616) (Figure 3A). The essential conditions for cell migration include cytoskeletal elements to form membrane ruff, lamellipodia and filopodia formation, and mitochondrial trafficking. Mitochondria provides the necessary energy supply for lamellipodia and filopodia formation during cancer cell migration [28]. To determine the effect of HMGB1 on mitochondrial trafficking, A549 cells transfected with LV-HMGB1 or si-HMGB1 were stained for mitochondria, nucleus, and α-tubulin using immunocytochemistry (ICC), and then they were analyzed using confocal microscopy and the Imaris software. LV-HMGB1 promoted mitochondrial trafficking, and the mitochondria were distributed along F-actin (Figure 3B) and α-microtubule (Figure 3C) to the lamellipodia leading edge and around the nucleus. In contrast, the knockdown of HMGB1 increased mitochondria assembly in the cytoplasm. The LV-HMGB1 group showed an increased amount of filopodia and branched filopodia formation compared with other groups. To verify filopodia formation through F-actin polymerization, the F-actin and G-actin ratios were analyzed by Western blot (WB) (Figure 3D). Our experimental results indicated that the LV-HMGB1 group had an increased F-actin polymerization, which contributed to filopodia formation. To investigate the effect of HMGB1 on cancer cell migration, a wound healing assay was performed (Figure 3E,F). HMGB1-mediated cell migration displacement, velocity, and motility tracks were examined and quantified using time-lapse confocal microscopy (Figure 3G–I). Our data showed that HMGB1 significantly increased lung cancer cell migration and motility.

### 2.4. HMGB1 Regulates Membrane Tension, Dynamics, Polarization, and Cytoskeletal Rearrangements in Lung Cancer Cells

**Figure 4:**
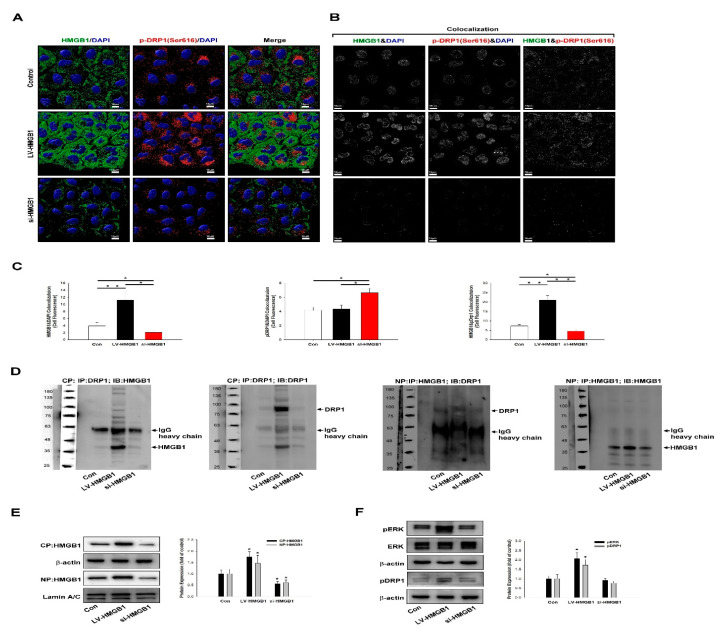
HMGB1 regulates DRP1 phosphorylation and colocalization. A549 cells were transfected with LV-HMGB1 or si-HMGB1 for 48 h. (**A**) Cells were stained with HMGB1 (green color), pDRP1 (Ser616) (red color), and nucleus dye (blue color), and they were analyzed using confocal microscopy and the Imaris software. (**B**,**C**) To validate that HMGB1 regulates pDRP1 (Ser616) expression, confocal analysis, image construction, and quantification were done using the Imaris software. Data are representative of three independent experiments. (**D**) HMGB1 colocalization with pDRP1 (Ser616) was determined by co-immunoprecipitation. (**E**,**F**) The regulation pathway was examined by a Western blot assay. * *p* < 0.05 compared with the control. Con: control. CP: cytosolic protein; NP: nucleus protein; IP: immunoprecipitation; IB: immunoblotting; IgG: immunoglobulin G; Lamin A/C: nucleus protein internal control.

To further investigate the association between HMGB1 and DRP1 in regulating lung cancer migration, the expression and colocalization of HMGB1 (green color) and pDRP1 (Ser616) (red color) were analyzed using an immunostaining assay (Figure 4A–C). LV-HMGB1 increased HMGB1 expression in the cytoplasm and nucleus. It also increased pDRP1 (Ser616) expression and colocalization with HMGB1 in the cytoplasm. HMGB1 silencing reduced HMGB1 expression in the cytoplasm and nucleus. A co-immunoprecipitation assay was employed to confirm the colocalization between HMGB1 and DRP1 (Figure 4D). HMGB1 inhibition increased nuclear pDRP1 (Ser616), reduced cytoplasmic pDRP1 (Ser616), and showed colocalization. HMGB1 was shown to promote ERK-mediated mitochondrial DRP1 phosphorylation, thus enhancing chemoresistance through RAGE in colorectal cancer (CRC) [21]. A previous study also indicated that the ERK2 phosphorylation of DRP1 (Ser616) promotes mitochondrial fission and MAPK-driven tumor growth [29]. Our data also confirmed that HMGB1 overexpression increased ERK-mediated mitochondrial DRP1 phosphorylation (Figure 4E,F).

### 2.5. HMGB1 Promotes Tumor Growth and pDRP1 (Ser616) Expression In Vivo

**Figure 5:**
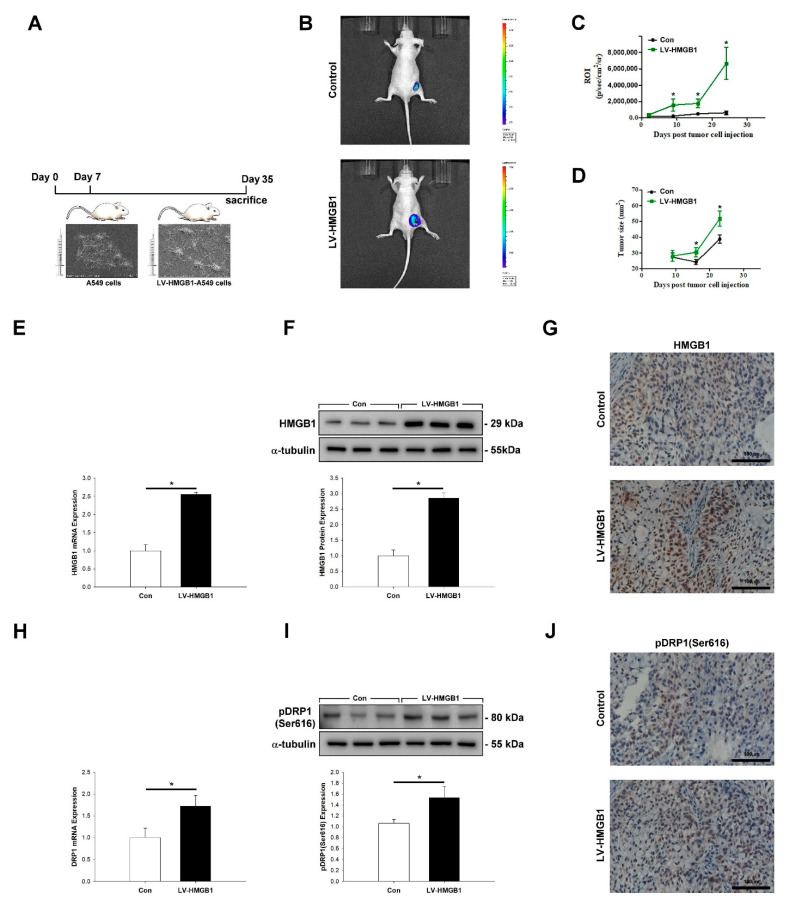
HMGB1 promotes tumor growth and pDRP1 (Ser616) expression in vivo. (**A**) Schematic diagram of the experimental process. Xenograft models were established using severe combined immunodeficiency (SCID) mice that were subcutaneously inoculated with the control or HMGB1-overexpressing (LV-HMGB1) A549 cells (*n* = 8 mice/group). (**B**) Fluorescent labeling of cells. (**C**,**D**) Fluorescence based on the region of interest and tumor size, respectively, were measured. (**E**–**G**) Expression of HMGB1 mRNA and protein in tumor tissues was assessed by real-time PCR, Western blotting, and an immunohistochemistry assay, respectively. (**H**–**J**) Expression of pDRP1 (Ser616) mRNA and protein in tumor tissues was measured by real-time qPCR, Western blotting, and an immunohistochemistry assay, respectively. * *p* < 0.05 compared with the control. ROI: region of interest.

To examine whether HMGB1 affected tumorigenic potential in lung cancer, A549 cells transfected with LV-HMGB1 and luciferase were subcutaneously injected into nude mice (Figure 5A). Tumor growth was monitored using an in vivo imaging systems (IVIS) (Figure 5B–D). HMGB1 and DRP1 expression in tumor tissues were evaluated using qPCR (Figure 5E–H), WB (Figure 5I), and immunohistochemistry (IHC) (Figure 5J). Our experimental results demonstrated that LV-HMGB1 enhanced tumor growth and increased pDRP1 expression.

### 2.6. Elevated Expression of HMGB1 and DNM1L Genes Predicts Poor Outcome in NSCLC

**Figure 6:**
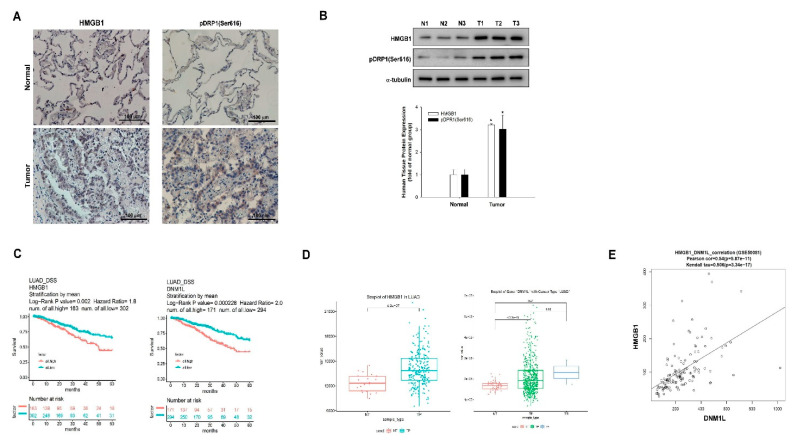
Elevated expression of *HMGB1* and *DNM1L* (*DRP1*) genes is associated with a poor outcome in NSCLC. (**A**,**B**) Expression of HMGB1 and pDRP1 (Ser616), respectively, in human normal or tumor tissues was examined by immunohistochemistry (IHC) and WB. * *p* < 0.01 compared with the control. (**C**) Effects of *HMGB1* and *DNM1L* expression on overall survival in lung cancer patients were analyzed; the Kaplan–Meier plots were generated using a Kaplan–Meier Plotter obtained from the DriverDBv3 database (NT, TP, and TR) (http://driverdb.tms.cmu.edu.tw/) [30]. (**D**) Correlations between *HMGB1* and *DNM1L* gene expression and 5-year overall survival rate of patients with lung cancer were obtained using the DriverDBv3 database. (**E**) Correlations of *HMGB1* and *DNM1L* expression, the Pearson’s correlation, and Kendall’s tau coefficient tests were used to calculate the *p* value. NSCLC: non-small-cell lung cancer; N: normal lung tissue; T: lung tumor tissue.

To confirm our results in non-small cell lung cancer (NSCLC) samples, IHC and WB analyses demonstrated that the expression of HMGB1 and DRP1 was significantly higher in tumor tissues than in normal lung tissues (Figure 6A,B). We also analyzed the expression of HMGB1 and DRP1 in NSCLC using the DriverDBv3 database [30] (tumor parts (TP), tumor recurrent (TR) tissues, and normal tissues (NT)). The high expression of HMGB1 (log-rank, *p* = 0.0002) and DRP1 (log-rank, *p* = 0.000228) correlated with a poor survival (Figure 6C). Both genes were highly expressed in TP and TR tissues compared with NT (HMGB1: *p* = 4.2 × 10^−7^; DRP1: *p* ≤ 2.22 × 10^−16^) (Figure 6D). In addition, the expressions of both HMGB1 and DRP1 revealed positive correlations in lung adenocarcinoma (Pearson correlation coefficient = 0.54, *p* = 5.87 × 10^−11^; Kendall tau = 0.506; *p* = 3.34 × 10^−17^) (Figure 6E).

## 3. Discussion

The metastatic process is a multi-step phenomenon involving cancer cell transitions with different polarized phenotypes [31]. Previous reports have shown that HMGB1 adhered to fibrinogen and induced the polarization of the myelomonocytic cell line [32]. This change in shape led to F-actin enrichment at the leading edge and indicated HMGB1-induced lamellipodium formation in endothelial [33] and inflammatory cells [32,34]. A previous study indicated a mechanism for length-dependent microtubule depolymerization, wherein polarized dynamic microtubules promoted cell elongation and spreading by changing the long cell axis towards cell tips [35]. In this study, we investigated the role of HMGB1 in phenotype transitions in lung cancer cells. Our results demonstrated that HMGB1 reduced cell membrane tension and increased the membrane area and lamellipodia/filopodia formation. Therefore, HMGB1 plays an important role in modulating cell polarization and motility.

Filopodia are “finger-like” plasma membrane protrusions that are made of ≥10 tightly bundled actin filaments [36]. These filaments are organized in a parallel manner by small crosslinking proteins, such as fascin, and their barbed ends face the plasma membrane [15]. The polarized nature of the actin filaments allows motor proteins to actively transport cargoes to slender protrusions [37]. The tips of the filopodia are dense and contain many proteins, including integrins [38]. It is unknown whether the filopodia tips also function as platforms for integrin outside–in signaling [39]. However, this is an intriguing possibility and might underlie the important role of filopodia in cell migration. The role of HMGB1 in filopodia formation during cancer metastasis has not been well-investigated. Understanding the mechanism by which HMGB1 regulates the interplay between actin and microtubule cytoskeleton during cancer metastasis is a critical issue. Our experimental results showed that HMGB1 modulated microtubule reorganization, such as F-actin polymerization, to form filopodia in the lamellipodia leading edge. Furthermore, HMGB1 regulated DRP1 phosphorylation and increased mitochondrial fission and trafficking through the actin and tubulin cytoskeletal system to the filopodia leading edge in order to supply the energy for cancer cell migration. To the best of our knowledge, this is the first study that explored the role of HMGB1 in filopodia formation in terms of driving migration in lung cancer cells. However, the study had limitations, and whether HMGB1 is a key regulator in modulating cortical F-actin and microtubule dynamics requires further confirmatory analysis.

To achieve local ATP production, mitochondria accumulate at regions of high-energy demand [19]. Therefore, docking and anchoring mechanisms are needed for the correct distribution of mitochondria. The roles of actin filaments and microtubules in mitochondrial transport have been extensively studied [40]. Metastatic breast cancer cells were found to enhance mitochondrial fission, increased DRP1 expression, and decreased mitofusin-1 expression [26]. Our experimental results also indicated that the overexpression of HMGB1 increased mitochondrial fission, promoted mitochondrial transportation to the filopodia leading edge by actin filaments and microtubules, and increased cell migration and motility. Moreover, the results of lung cancer xenograft animal model confirmed that the overexpression of HMGB1 promotes tumor growth and increases DRP1 expression and phosphorylation in the nucleus and cytoplasm. In lung cancer, no associated literature has investigated the association between HMGB1 and DRP1 until now. We demonstrated that DRP1 accumulates in the nucleus and cytoplasm in the LV-HMGB1 group. HMGB1 significantly increased the phosphorylation of DRP1 in both the nucleus and cytoplasm and colocalized with HMGB1. However, the role of colocalization between HMGB1 and DRP1 in lung cancer migration is still unknown and needs further investigation.

A recent study showed that HMGB1 promoted RAGE-mediated chemoresistance through ERK-activated mitochondrial DRP1 phosphorylation in CRC [21]. Our results also showed that HMGB1 increased ERK and DRP1 phosphorylation (Ser616). A previous study indicated that nuclear DRP1 is mostly phosphorylated under hypoxia, whereas the intracellular distribution of DRP1 is essential for determining the drug sensitivity of lung adenocarcinomas, suggesting that the expression of DRP1 is associated with survival in patients [41,42]. In addition, HMGB1 exerts autocrine or paracrine effects that activate DRP1 and lead to tumor recurrence [21]. Our experimental results indicated that patients with high levels of DRP1 demonstrated a significant correlation with poor five-year survival rates and have a high risk of tumor recurrence. Using the DriverDBv3 database analysis, we found that high expressions of HMGB1 and DRP1 were associated with a poor prognosis, and the genes were positively correlated [30].

## 4. Materials and Methods

### 4.1. Cell Culture

The human lung cancer cell line, A549, was obtained from Bioresource Collection and Research Center (BCRC; Hsinchu, Taiwan), and confirmed by the short tandem repeat-PCR profile at BCRC (D7S820: 8, 11 CSF1PO:10, 12 TH01: 8, 9.3 D13S317: 11 D16S539: 11, 12 vWA: 14 TPOX: 8, 11 amelogenin: X, Y D5S818:11). A549 cells were cultured in an F-12K growth medium (Thermo Fisher Scientific, Waltham, MA, USA) supplemented with 5% fetal bovine serum (Invitrogen, Carlsbad, CA, USA), 100 units/mL of penicillin, and 100 pg/mL of streptomycin (Sigma-Aldrich, St. Louis, MO, USA) at 37 °C in a humidified atmosphere of 95% air–5% CO_2_. Culture media were replaced every 4–5 days, and the cells were subcultured 3–12 times for experiments.

### 4.2. Cell Migration and Motility Assays

IBIDI™ Culture Inserts (IBIDI, Martinsried, Germany) were placed into 6-well culture dishes, and 1 × 10^4^ cells/mL were seeded into the culture-inserts in the 35 mm μ-Dish, which could be used as two reservoirs of the same insert [43]. After 24 h, the insert was carefully removed to create a 0.5-mm gap, and cell migration was assessed using bright-field microscopy at 0 and 24 h after insert removal. The migrated cells were imaged, and the covered areas were measured using the WimScratch software program (Wimasis, Munich, Germany). Cell motility, tracks, displacement, and speed were assessed using time-lapse confocal microscopy (SP2; Leica, Exton, PA, USA) at various time points over a 20 h period.

### 4.3. qPCR

Total RNA (2 μg) was reverse-transcribed using the SuperScript First-Strand Synthesis System (Invitrogen). In accordance with the minimum information for publication of qPCR experiment guidelines [43], the analysis was performed using a TaqMan assay (LightCycler FastStart DNA master SYBR green I; Roche, Basel, Switzerland). The following primers were used: the human HMGB1 sense primer 5′-ATT CAA GGA TCC CAA TGC AC-3′ and the antisense primer 5′-GAT TTT TGG GCG ATA CTC AGA-3′; the human DRP1 sense primer 5′-GCG CTG ATC CCG CGT CAT-3′ and the antisense primer 5′-CCG CAC CCA CTG TGT TGA-3′; and the human GAPDH sense primer 5′-AGC CAC ATC GCT CAG ACA-3′ and the antisense primer 5′-GCC CAA TAC GACCAA ATC C-3′ (Genomics, New Taipei City, Taiwan).

### 4.4. WB Assay

The experiments were performed as described previously [44]. The protein concentrations of cell and tissue lysates were measured using the Lowry assay, and 30 µg of total protein were separated by SDS-PAGE in 7.5, 10, or 12.5% gels depending on the molecular weight of the target. Primary antibodies against HMGB1 (1:1000; 66525-1-Ig, Proteintech, Rosemont, IL, USA), pDRP1(Ser616) (1:500; AP0849, ABclonal, MA, USA), pERK (1:500, #9101, Cell Signaling, Beverly, MA, USA), ERK (1:500; sc-135900, Santa Cruz Biotechnology, CA, USA), α-tubulin (1:2000; T5168, Sigma, Louis, MO, USA), Lamin A/C (1:1000, GTX101127, GeneTex, Irvine, CA, USA), and α-actin (1:2000, sc-137179, Santa Cruz Biotechnology) were used.

### 4.5. Construction of a Human HMGB1 Lentiviral Vector and Cell Transduction

For the cloning of human HMGB1 (GENE ID:3416, GenBank), total RNA was isolated from A549 cells, and cDNA was synthesized using Moloney murine leukemia virus reverse transcriptase according to the manufacturer’s protocol (Invitrogen). The human HMGB1 lentiviral vector was constructed, and cell transduction was performed as described previously [12]. The recombinant clones stably expressing human HMGB1 were selected using 1 mg/mL of puromycin.

### 4.6. HMGB1 Silencing

A549 cells were seeded in 6-well plates for 24 h, transfected with 20 nM of human HMGB1 si-RNA (si-HMGB1) (AM16706, Invitrogen) using the Lipofectamine 3000 transfection reagent (Invitrogen), and analyzed after 48 h.

### 4.7. FE-TEM

FE-TEM was performed as described previously [43]. Briefly, cells were fixed with 2.5% glutaraldehyde for 2 h at 4 °C, washed, post-fixed in 1% osmium tetroxide for 2 h, dehydrated in graded acetone, infiltrated, and embedded in epoxy resin. Ultrathin 70 nm sections were cut using a Leica RM2165 microtome (Leica RM2165, Japan) and examined under an FE-TEM microscope (HITACHI HT-7700, Japan) at an accelerating voltage of 80 kV.

### 4.8. FE-SEM

FE-SEM was performed as described previously [43]. Cultured cells were seeded on 0.17 mm-thick cover slips and fixed in 2.5% glutaraldehyde overnight at 4 °C, post-fixed in 2% osmium tetroxide for 1.5 h at 4 °C, and dehydrated in ascending grades of alcohol (50%, 75%, 85%, 95%, and 100%). Samples were dried using a critical point dryer (CPD 030, Bal-TEC) for 1 h, coated in gold, and examined under FE-SEM (Hitachi-8010, Japan) at an accelerating voltage of 10–25 KV.

### 4.9. Measurement of F-Actin/G-Actin Ratio

The ratio of F-actin to G-actin was determined using the G-actin/F-actin in vivo assay kit (BK037, Cytoskeleton, Denver, Colorado) according to the manufacturer’s instructions. Briefly, 100 μg of protein from A549 cells were homogenized in lysis and an F-actin stabilization buffer and centrifuged at 2000 rpm for 5 min to remove unbroken cells. F-actin was separated from G-actin by centrifugation at 100,000× *g* for 60 min at 37 °C. The F-actin-containing pellet was resuspended in an F-actin depolymerizing buffer at a volume equivalent to the G-actin-containing supernatant volume. The resuspended F-actin pellet was kept on ice for 60 min and gently mixed every 15 min to dissociate F-actin. Proteins in equivalent volumes (20 μL) of supernatant and pellet were separated by SDS-PAGE and subjected to immunoblot analysis using an anti-pan actin antibody supplied in the kit. The F/G actin ratio was quantified using the ImageJ software (NIH, MD, USA).

### 4.10. Tumorigenic Potential of HMGB1-Overexpressing A549 Lung Cancer Cells

Animal studies were performed as described previously [44]. Male (6–8-week-old) severe combined immunodeficiency (SCID) mice purchased from BioLasco Company (Taipei, Taiwan) were housed in a special pathogen-free room with a 12-h light/12-h dark cycle and 40–70% humidity at 19–25 °C, and they had free access to a standard rodent diet and water ad libitum. Before inoculation, cells were infected with lentiviruses carrying luciferase. Mice (n = 8 per group) were subcutaneously inoculated in the flanks with 0.1 mL of PBS containing 1 × 107 of the control or HMGB1-expressing A549 cells and monitored for bioluminescence intensity during tumor growth twice a week for 25 days using the IVIS (Xenogen, Alameda, CA, USA). Tumor volume was calculated as V (mm^3^) = length (L, mm) × width (W, mm^2^) × 0.5. The protocol for the animal study was approved by the Department Center for Biotechnology (DCB) Institutional Animal Care and Use Committee (Approval No:102050) (Taipei, Taiwan).

### 4.11. Tumor Sample Collection and DriverDBv3 Database Analysis

NSCLC samples and corresponding normal tissues were collected from 48 non-selected patients who underwent surgical resections at the Division of Thoracic Surgery, Department of Surgery, Kaohsiung Medical University Hospital from 2004 to 2007. Lung tumors were classified by histological type, grade, and stage according to the World Health Organization standards [45]. Informed consent was obtained from patients to participate in the study. The study was approved by the Ethical Review Board for Research (KMUH-IRB-940292 and KMUH-IRB-950276) in Kaohsiung Medical University Hospital, Taiwan. The effects of HMGB1 and DRP1 (dynamin 1-like (DNM1L)) expression levels on overall survival in patients with lung cancer were analyzed; the Kaplan–Meier plots were generated with the aid of a Kaplan–Meier Plotter using the DriverDBv3 database (tumor parts (TP), tumor recurrent (TR) tissues, and normal tissues (NT)) (http://driverdb.tms.cmu.edu.tw/) [30]. The correlation of gene expression was analyzed using Pearson’s correlation and Kendall’s tau tests.

### 4.12. IHC Analysis

For the protein expression analysis of HMGB1 and DRP1, 5 µm-thick paraffin tissue sections or cultured cells were incubated in a blocking buffer (0.5% bovine serum albumin and 0.05% Tween 20 in PBS) for 1 h at room temperature (RT). Then, the sections or cells were incubated with primary antibodies against HMGB1 (1:200) and DRP1 (1:100) for 1 h, stained, and visualized using a fluorescence detection system (Ventana Medical Systems, Invitrogen). Samples were counterstained with 4′,6-diamidino-2-phenylindole (DAPI; Invitrogen) to visualize cell nuclei. After washing, sections were mounted in VECTASHIELD mounting medium (Invitrogen) and examined under a confocal laser microscope (Leica, Miami, FL, USA).

### 4.13. ICC Analysis

ICC was performed as described previously [44]. Briefly, cells were cultured on glass coverslips, washed with cold PBS, and fixed with 4% paraformaldehyde in PBS at 4 °C for 15 min. After blocking, cells were incubated with the primary antibodies against HMGB1 (1:200), pDRP1 (1:100), α-tubulin (1:500), or F-actin (1:500; AB205, Abcam, Cambridge, MA, USA) overnight at 4 °C, rinsed with PBS, and incubated with rhodamine-conjugated secondary antibodies for 1 h at RT; cell nuclei were stained with DAPI. After washing with PBS, cells were mounted in a VECTASHIELD mounting medium and analyzed using an Olympus FV1000 confocal laser scanning microscope (Center Valley, PA, USA) and the Imaris software (OXFORD instruments, Concord, MA, USA) for confocal image 3D reconstruction.

### 4.14. Co-Immunoprecipitation Assay

The assay was performed as described previously [46]. To determine the protein–protein interactions between HMGB1 and DRP1, cells were harvested using an immunoprecipitation lysis buffer (GeneTex). Total proteins (500 μg) were purified by incubation with protein A/G agarose beads (50 μL/tube), under rotating conditions, for 1 h at 4 °C, and washed. The supernatants were collected and incubated with 2 μg of anti-HMGB1 and anti-DRP1 antibodies for 4 h. Protein A/G agarose beads (50 μL/tube) were added to each tube, followed by overnight incubation at 4 °C. The supernatants were removed by centrifuging at 12,000× *g* for 10 min and disrupted by boiling in 1% SDS. The immune complexes were analyzed by immunoblotting with anti-HMGB1 and anti-DRP1 antibodies.

### 4.15. Statistical Analyses

The data are presented as means ± SEM and were statistically analyzed by the analysis of variance and Dennett’s test using SigmaStat version 3.5 (Systat Software Inc., San José, CA, USA). A *p* value > 0.05 was considered to be statistically significant.

## 5. Conclusions

In summary, we demonstrated the significant role of HMGB1 in regulating lung cancer migration. Our study showed that HMGB1 increased lung cancer cell spreading and polarization, regulated microtubule and actin dynamics to cell protrusions, and promoted lamellipodia and filopodia formation. Consequently, HMGB1 increased DRP1 expression, phosphorylation, and colocalization. In addition, DRP1 phosphorylation mediated mitochondrial fission and trafficking to the lamellipodia and filopodia leading edge by actin and microtubule cytoskeleton to supply energy for lung cancer migration.

## Data Availability

Not applicable.
